# Bureau of Mines Method of Calibrating a Primary Radon Measuring Apparatus

**DOI:** 10.6028/jres.095.012

**Published:** 1990

**Authors:** R. F. Holub, W. P. Stroud

**Affiliations:** U.S. Department of the Interior, Bureau of Mines, Denver, CO

**Keywords:** calibration, measurement, radon

## Abstract

One important requirement for accurate monitoring of radon in working environments, dwellings, and outdoors is to ensure that the measurement instrumentation is properly calibrated against a recognized standard. To achieve this goal, the U.S. Department of Interior Bureau of Mines (BoM) Radiation Laboratory has participated since 1983 in a program to establish international radon measurement standards. Originally sponsored by the Organization for Economic Cooperation and Development (OECD), the program is also sponsored by the International Atomic Energy Agency. While the National Institute of Standards and Technology (NIST) radium solution ampules are acceptable to all participating laboratories as a primary standard, a method of transferring radon from the NIST source into each laboratory’s primary counting apparatus is a critical problem. The Bureau’s method transfers radon from the primary solution by bubbling 3 L of air through it into a steel cylinder. After homogenizing the radon concentrations in the cylinder, eight alpha-scintillation cells are filled consecutively and measured in a standard counting system. The resulting efficiency is 81.7±1.2%.

## 1. Introduction

A major part of the U.S. Department of Interior Bureau of Mines (BoM) radiation hazard program has been the establishment of standards for the measurement of radon and radon daughters, both for the Mine Safety Health Administration and private industry. Consistent with this objective, the Bureau was invited to participate in a cooperative effort to establish international measurement standards. The first publication of the results of this program [1], Part 1, Radon Measurement, pointed out that there is a 7% disagreement among the four participating laboratories and that further work is needed. These discrepancies were also reported by the U.S. Department of Energy (DoE) Environmental Measurements Laboratory [2]. Surprisingly, this disagreement was largely unnoticed even as late as 1988 [3].

## 2. Description of the Method

**Figure 1:**
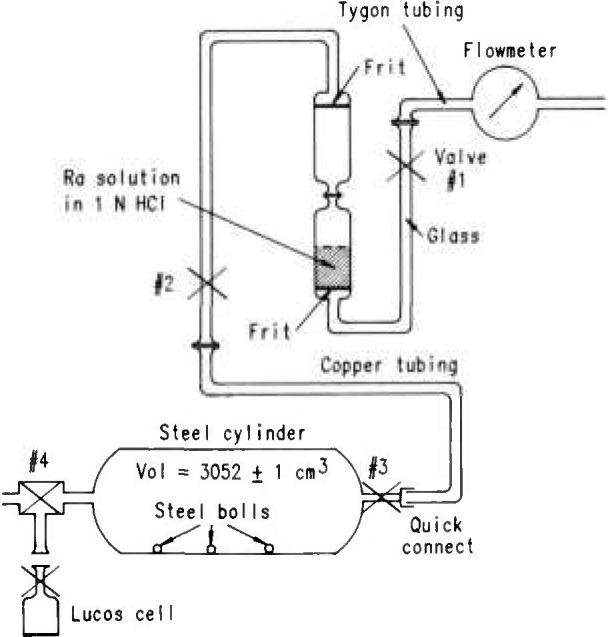
Schematic of the BoM method of calibrating a primary radon measuring apparatus.

**Figure 2:**
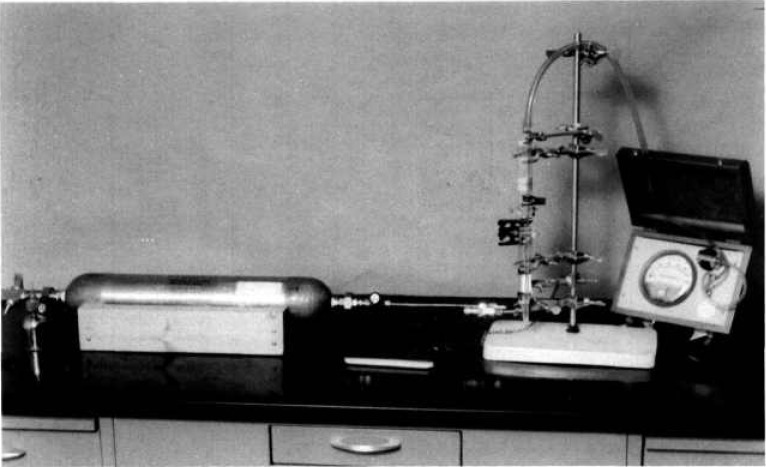
Photograph of the BoM system.

Figure 1 shows the schematic of the Bureau’s system. Figure 2 is a photograph of the system comprising the de-emanation flask, the connecting tubing and a steel cylinder for the transferred radon.

The first step in the procedure is to evacuate the 3052 ±1 cm^3^ steel cylinder and connect it to the radium solution/frit system. The inlet to this system has a flowmeter to monitor the flow rate. Control of the flow is accomplished by carefully operating valve #1, upstream from the solution. The second valve is opened all the way for the transfer operation.

After the radon gas is collected in the cylinder, the cylinder is disconnected and shaken vigorously for several minutes so that five steel balls inside homogenize the gases, ensuring a uniform concentration. It has been shown that failure to homogenize results in large errors.

**Figure 3:**
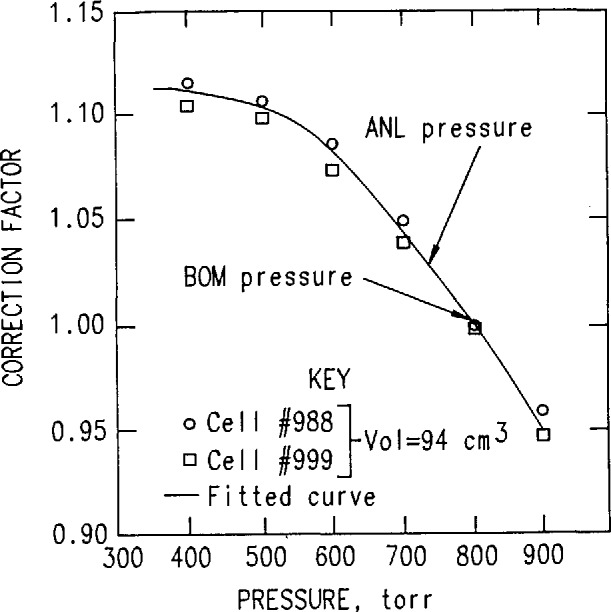
Pressure correction as a function of pressure inside the cell. The cells have a Ra source embedded in the window.

**Figure 4:**
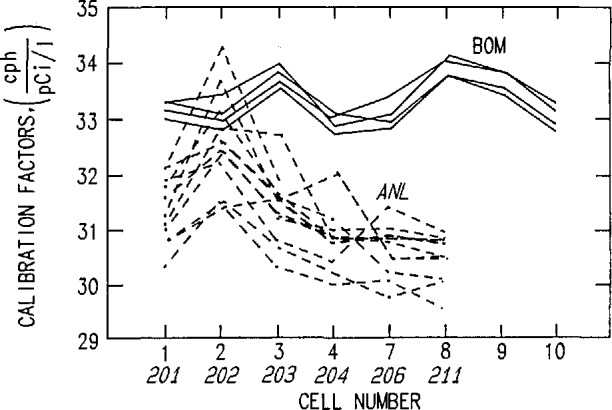
Plot of the BoM and ANL results. BoM and ANL cells have 102 and 92 cm^3^ volumes, respectively.

Evacuated alpha-scintillation cells (commonly known as Lucas cells) are then connected to the cylinder and samples taken of the radon-containing air using valve #4; each consecutive sampling is corrected for the diminishing concentration of radon in the steel cylinder. Cells are then pressurized to 800 Torr, a standard method adopted at the Bureau to avoid the need to make pressure corrections. The magnitude of this correction is shown in figure 3. The results for four consecutive runs (32 hourly measurements in total), the efficiencies, and the calibration factors are given in table 1 and figure 4. In addition to the BoM results, the results reported by Lucas [4] from Argonne National Laboratory (ANL) are also shown in figure 4. Note that the BoM cell volume is 102 cm^3^, whereas that used by Lucas is 92 cm^3^.

## 3. Experimental

### 3.1 Determination of Cylinder Volume

A stainless steel cylinder approximately 53 cm in length and 10 cm in diameter was fitted at both ends with high-vacuum valves, as shown in figure 1. The five small steel balls of approximately 1.5-cm diameter were placed in the cylinder to facilitate mixing of the radon/air mixture. Volume was determined from valve seat to valve seat at opposite ends of the cylinder.

All procedures in this report were carried out at the normal ambient laboratory temperature of 21 °C. Accordingly, a nominal 1,000 mL burette was calibrated at 21 °C by weight of water delivered. Three successive determinations gave an average value of 996 mL delivered water per 1,000 mL indicated. Maximum deviation between the three calibrations was 1 mL. Water density at 21 °C was assumed to be 0.998 g/mL.

The stainless steel cylinder was then evacuated, the three-way #4 valve was closed, and the cylinder placed vertically beneath the calibrated burette. A length of 3/8-in Tygon^1^ tubing was attached to the burette tip. The stopcock of the filled burette was opened, and water allowed to flow through the tubing until no air bubbles remained. The water-filled tube was then connected to the nipple of the valve. The unevacuated volume of this nipple was measured to be less than 1 mL. The burette was then filled to the 1,000 mL-mark, and the entire system allowed to equilibrate to 21 °C.

The three-way valve #4 and valve #3 were opened, and the evacuated cylinder allowed to fill with water. Transfer was made in increments of 1,000 mL. No change on the final burette reading was observed over a period of 1 h. Two successive determinations resulted in cylinder volumes of 3.051 and 3,052 mL. The final accepted value was 3.052 ml at 21 °C.

### 3.2 Transfer of Standard Reference Material

A Standard Reference Material, No. 4953D, was obtained from NIST. This source consisted of approximately 5 g of BaCl_2_ carrier solution in 1.4 *N* HCl. The certified Ra-226 concentration of the solution was 3.984 ×10^−9^ g of Ra-226 per g of solution. Transfer of the reference material to the de-emanation flask was greatly facilitated by “doll bottles” provided by Dr. Isabelle Fisenne of the DoE Environmental Measurements Laboratory. The doll bottles were small polyethylene flasks of some 7-mL capacity. The tips of these bottles were heated and drawn out to form capillary tubes some 10 cm in length.

The glass ampule containing the Ra-226 standard was opened, and the contents aspirated into a doll bottle via the capillary neck. Bottle and solution were then weighed to 0.01 mg, and the weight recorded. The Ra-226 solution was transferred quantitatively to a 100-mL de-emanation flask containing some 50 mL of 1 *N* HCl. The empty doll bottle was then reweighed to 0.01 mg. The actual amount of solution transferred was 4.95229 g, resulting in a Ra-226 activity of 19.50 ×10^−9^ Ci. The de-emanation flask was then sealed, with the date and time noted.

### 3.3 De-emanation and Transfer to the Cylinder

Air volume in the sealed de-emanation flask had previously been determined to be about 100 mL. Filling of the 3,052-mL evacuated cylinder then represented roughly 30 transfers of equilibrium vapor. Using a solubility coefficient, *k*, of 0.25 [5], and *n* equal to 30, the fraction of radon remaining in the liquid phase after filling the cylinder is given by the expression

$$Rn(\text{liquid})/Rn(\text{vapor})={\left(1-k\right)}^{n}$$

where *n* equals the number of transfers of the vapor phase. Passage of some 3,000 mL of air through the de-emanation flask represents a transfer of over 99% of the equilibrium radon atoms. Previous experiments had determined that a flow rate of 100 mL/min should not be exceeded in order to prevent any possible liquid phase transfer to the steel cylinder. To further minimize any possibility of this occurrence, high efficiency glass filters were placed in the fittings at both ends of the cylinder. Accordingly, the steel cylinder was evacuated to 630 Torr, and the three-way valve closed. The cylinder was then connected to the de-emanation flask as shown in figure 1. The cylinder valve and the de-emanation flask stopcock were opened and the date and time recorded. Flow rate was monitored by means of the flowmeter at the entry port of the de-emanation flask. At the end of 43 min, a small, positive flow rate of about 3 mL/min was still observed. At this time, both valve and stopcock were closed, date and time recorded, and the cylinder disconnected from the de-emanation flask.

### 3.4 Loading and Counting the Cells

Evacuated cells were loaded through the three-way valve #4 (fig. 1) and pressurized to 800 Torr. The radon-free air used for pressurization was taken from an ordinary steel cylinder determined to be free of Ra contamination. The pressurization was measured in each cell to ±1 Torr. The pressurized cells were counted overnight at 1-h intervals using conventional photomultiplier tubes and counting equipment. The measured activity was extrapolated to time zero using a radon half life of 3.8235 days. Results were recorded and the standard deviation δ was calculated.

## 4. Discussion

Results, summarized in table 1, show the overall efficiency to be 81.7±1.2% in contrast to the 84.6±1.9% that ANL reported. Theoretical counting errors were calculated to range between 0.34% to 0.48%, as opposed to the observed values appearing in the last column of table 1.

A list of possible errors, summarized in table 2, includes:

1. Loss of material during the transfer from the doll bottle into the de-emanation flask. This error would result in a lower calibration coefficient.
2. Weighing errors during transfer of material. This error could result in either positive or negative deviation.
3. Incomplete transfer of radon into the steel cylinder. It seems that this could happen only if radon in the cylinder streamed against the flow into the system—an unlikely case. Both this error and error number 1 would lead to smaller calibration factors and efficiencies—exactly the opposite direction from that needed to bring the Bureau’s results into agreement with the other laboratories [1]. On the other hand, a 2% to 4% loss could explain the Bureau’s difference from Argonne National Laboratory results [4].
4. Pressure deviations in the Lucas cell could cause errors. The pressure correction curve, shown in figure 3, was obtained by using two cells that had a radon source incorporated into their window. Note that at Denver’s elevation, the error is about +6%.
5. Errors in determination of the volume of the steel cylinder.
  There are two cases:
  - The assumed volume would be greater than the true volume, leading to a lower calculated efficiency. However, the error would have to be about +60 cm^3^ compared to our stated error of ± 1 cm^3^, thereby excluding this possibility.
  - The assumed volume would be smaller than the true value. This is more likely than 5a. The presence of an unrecognized volume somewhere in the system might explain, for instance, why the ANL intercomparison results are lower than the Bureau’s.
6. Timing error is not very significant because even a 20 min error in timing corresponds to only 0.25% of the time-zero count rate.
7. Differences among cells because of variations in photon yields from alpha-particle bombardment of the ZnS layer. This error probably accounts for the systematic variations shown in figure 4. It is not clear how to deal with these errors without spending a great deal of effort in perfecting the Lucas cells.
8. Different plateout characteristics on the ZnS-covered surfaces vs the window surface of Lucas cells. The role of natural convection due to temperature differences between inside air and the walls was investigated [6] and found to be a possible source of error. To determine the magnitude of this error would require a great deal of effort, also perhaps not justified in view of the fact the error is believed to be relatively small.

## 5. Conclusions

The Bureau’s method of transferring radon from NIST solution into Lucas cells provides a reliable and relatively permanent system for primary calibrations. The recognized systematic errors in this method appear to be in the l%–2% range.

## Figures and Tables

**Table 1:** Efficiencies for two runs, each repeated twice

Cell					Average	%SD
1	0.8097	0.8168	0.8165	0.8139	0.8142	0.40
2	0.8053	0.8201	0.8118	0.8094	0.8117	0.76
3	0.8228	0.8342	0.8299	0.8260	0.8282	0.60
4	0.8025	0.8064	0.8118	0.8088	0.8074	0.49
7	0.8050	0.8122	0.8077	0.8186	0.8109	0.73
8	0.8277	0.8378	0.8279	0.8352	0.8322	0.62
9	0.8196	0.8298	0.8223	0.8295	0.8253	0.62
10	0.8027	0.8156	0.8053	0.8126	0.8091	0.75

Total average=					0.817	±1.2%

**Table 2:** Possible errors

	Effect on calibration coefficients		Remarks
	Higher	Lower	
Incomplete transfer of radium into flask		×	Not observed
Weighing error	×	×	
Incomplete transfer of Rn into cylinder		×	Back flow eliminated by having positive flow at all times
Pressure deviations in Lucas cell	×		Overpressurizing minimizes this error
Errors in cylinder volume	×	×	Successive experiments show ± 1 cm^3^ repeatability
Timing error	×	×	Considered insignificant
ZnS sulfide coating differences	×	×	Difficult to determine and eliminate
Changing plateout	×	×	Difficult to determine and eliminate

## Footnotes

1 Certain commercial equipment, instruments, or materials are identified in this paper to specify adequately the experimental procedure. Such identification does not imply recommendation or endorsement by the National Institute of Standards and Technology, nor does it imply that the materials or equipment identified are necessarily the best available for the purpose.
